# Antimicrobial-Resistant *Shigella* spp. in San Diego, California, USA, 2017–2020

**DOI:** 10.3201/eid2806.220131

**Published:** 2022-06

**Authors:** Thaidra Gaufin, Jill Blumenthal, Claudia Ramirez-Sanchez, Sanjay Mehta, David T. Pride, Joshua Fierer, Jeffrey D. Jenks

**Affiliations:** Sansum Clinic Infectious Diseases, Santa Barbara, California, USA (T. Gaufin);; University of California San Diego, La Jolla, California, USA (J. Blumenthal, C. Ramirez-Sanchez, S. Mehta, D.T. Pride, J. Fierer);; San Diego Veterans Affairs Medical Center, San Diego (S. Mehta, J. Fierer);; Durham County Department of Public Health, Durham, North Carolina, USA (J.D. Jenks)

**Keywords:** Shigella sonnei, Shigella flexneri, antibiotic resistance, antimicrobial resistance, AMR, men who have sex with men, MSM, GBM/transgender, unhoused, methamphetamine use, infectious diarrhea, bacteria, San Diego, California, USA

## Abstract

Annually, *Shigella* spp. cause ≈188 million cases of diarrheal disease globally, including 500,000 cases in the United States; rates of antimicrobial resistance are increasing. To determine antimicrobial resistance and risk factors in San Diego, California, USA, we retrospectively reviewed cases of diarrheal disease caused by *Shigella flexneri* and *S. sonnei* diagnosed during 2017–2020. Of 128 evaluable cases, *S. flexneri* was slightly more common than *S. sonnei*; most cases were in persons who were gay or bisexual cisgender men, were living with HIV, were unhoused, or used methamphetamines. Overall, rates of resistance to azithromycin, fluoroquinolones, ampicillin, and trimethoprim/sulfamethoxazole (TMP/SMX) were comparable to the most recent national data reported from the Centers for Disease Control and Prevention; 55% of isolates were resistant to azithromycin, 23% to fluoroquinolones, 70% to ampicillin, and 83% to TMP/SMX. The rates that we found for TMP/SMX were slightly higher than those in national data.

*Shigella* bacteria are facultatively anaerobic, nonmotile, gram-negative bacilli and a common global cause of infectious diarrhea, especially in low-income settings (*1*). They are relatively resistant to the high acidity of the stomach and can survive transit into the small intestine, where they multiply and pass into the colon, invading colonic cells and causing diarrhea. As few as 10–100 organisms are capable of causing disease (*2*). This low infectious dose increases transmission by the fecal–oral route or via contaminated food and water. Globally, *Shigella* spp. cause an estimated 188 million cases of diarrheal disease annually and account for 164,000 deaths (*1*), particularly in children.

In the United States, ≈500,000 cases of shigellosis occur annually; 81% are caused by *Shigella sonnei*, followed by *S. flexneri* (12.6%), *S. boydii* (0.2%), and *S. dysenteriae* (0.1%) (*3*). Most transmission occurs via the fecal–oral route, at locations such as daycare centers, but transmission can also occur after ingesting contaminated food or drinking water, after ingesting untreated recreational water (*4*), and among men who have sex with men (MSM). Among MSM, multiple outbreaks have been described in Montreal, Quebec, Canada (*5*,*6*), and Germany (*7*); and in the United States (*8*,*9*), including Minnesota (*10*); the Chicago, Illinois, and Minneapolis-St. Paul, Minnesota, regions (*11*); and New York, New York (*12*).

For children and adults with symptomatic shigellosis, antimicrobial therapy is typically recommended because it shortens symptom duration and decreases the duration of bacterial shedding in adults (*13*) and children (*14*–*17*). For children, antimicrobial therapy should be guided by local resistance profiles. For adults, empiric antimicrobial therapy selection should be guided by local resistance profiles as well as patient demographics. For example, in parts of Asia, widespread resistance to azithromycin, ceftriaxone, and ciprofloxacin has been reported (*18*,*19*). In addition, increasing antimicrobial resistance has been found in certain groups, such as MSM, and has also been associated with travel (*20*), including increasing resistance to first-line agents such as fluoroquinolones (*5*,*7*) and azithromycin (*8*). Last, susceptibilities obtained from culture should be used in the event of treatment failure.

To determine antimicrobial resistance and risk factors in the San Diego, California, USA, area, we investigated cases of symptomatic shigellosis causing diarrheal disease at the University of California San Diego Health System and San Diego Veterans Affairs Medical Center over a 3-year period. The study was approved by the Human Research Protection Program at University of California San Diego Health and the San Diego Veterans Affairs Institutional Review Board.

## Methods

We analyzed the electronic health records (EHRs) of persons >18 years of age who received care at the University of California San Diego Health System or the San Diego Veterans Affairs Medical Center from March 1, 2017, through May 31, 2020, for diarrhea and a diagnosis of shigellosis. We did not include persons <18 years of age because those data were not accessible in our EHR.

Stool samples from study participants were cultured or tested initially with a multiplex PCR panel ordered by providers at the University of California San Diego Health System or the San Diego Veterans Affairs Medical Center. Isolates that were positive for *Shigella* spp. on multiplex PCR were confirmed by stool culture for *Shigella* spp. on matrix-assisted laser desorption/ionization-time of flight mass spectrometry. Those isolates were then sent to the San Diego County Public Health laboratory to confirm speciation.

For persons for whom *Shigella* spp. isolation from stool culture was confirmed, we collected further information through EHR review. Information included patient ethnicity, race, age at diagnosis, history of travel out of San Diego County within 14 days before diagnosis, housing status, recent methamphetamine use, HIV status, use of highly active antiretroviral therapy (HAART), or use of preexposure prophylaxis (PrEP) for HIV prevention.

The most frequently reported antimicrobial susceptibility panel for *S. flexneri* and *S. sonnei* includes ampicillin, a fluoroquinolone, and trimethoprim-sulfamethoxazole (TMP/SMX); thus, we included these antimicrobials in the comparative analysis. The specific fluoroquinolone reported at each site differed. Most were ciprofloxacin; however, other fluoroquinolones, such as moxifloxacin or levofloxacin, were reported for a few persons. For the purpose of comparative analysis, we grouped fluoroquinolones together and reported them as either sensitive or resistant; we identified intermediate strains as resistant. We used broth microdilution for susceptibility testing. The standards for resistance were set by the Clinical and Laboratory Standards Institute (CSLI, https://clsi.org). The standards changed in 2022 (*21*), when CSLI began including azithromycin susceptibility, but we did not use these latest guidelines for our study because our data included only time points until May 2020.

We analyzed the data by using SPSS Statistics 27 (IBM, https://www.ibm.com) and descriptive statistics via cross-tabulations with the Fisher exact test and χ^2^ by creating a 2-by-2 table comparing different variables: resistance of various antimicrobials cross-tabulated with HIV status, sexual orientation, use of HAART if living with HIV, use of PrEP, being unhoused, or recent use of methamphetamines. We included methamphetamine use because this information was readily available on chart review (based either on positive urine toxicology screen or documented recent use in physician notes). Data associated with other substance use on chart review were not available. We considered p<0.05 to indicate statistical significance.

## Results

Of the 140 EHRs for patients for whom *Shigella* spp. was isolated from >1 stool specimen, we included 128 in our analysis and excluded 12 (for 6, the *Shigella* isolate was not speciated; for 2, *S. boydii* was isolated from stool culture; for 2, there was not enough information to complete the analysis; and for 2, duplicate positive isolates were obtained within a 2-week period). Overall, 55% of *Shigella spp.* isolates were identified as *S. flexneri* and 45% as *S. sonnei*. Patient ages ranged from 15–79 years; mean age was 47 years (Table 1). Eighty-one percent were male. Most patients self-identified as non-Hispanic white. Fifty-six percent identified as gay or bisexual male (GBM), and 3 identified as transgender women who had sex with men. Fifty-one percent were living with HIV, among whom 85% were receiving HAART; for 11%, HIV status was unknown. Twenty-three percent were unhoused, and for 33%, recent methamphetamine use was documented.

**Table 1:** General characteristics of persons with diarrheal disease and *Shigella* spp. isolated from stool culture, San Diego, California, USA, March 1, 2017–May 31, 2020*

Characteristic	Frequency, no. (%)
*Shigella* spp. infection	
* S. flexneri*	71 (55)
* S. sonnei*	57 (45)
Sex	
M	104 (81.3)
F	21 (16.4)
Transgender, male-to-female	3 (2.3)
Ethnicity	
Hispanic/Latin	28 (21.9)
Mixed race/other	16 (12.5)
Native Hawaiian/Pacific Islander	1 (0.8)
Non-Hispanic Black	12 (9.4)
Non-Hispanic White	69 (53.9)
Unknown	2 (1.6)
Sexual orientation	
GBM/transgender	71 (55.5)
Not GBM	20 (15.6)
Unknown	37 (28.9)
HIV status	
Positive	65 (50.8)
Negative	49 (38.3)
Unknown	14 (10.9)
HAART use by persons living with HIV	
Yes	55/65 (84.6)
No	10/65 (15.4)
PrEP use among persons at risk for HIV	
Yes	8/12 (66.7)
No	4/12 (33.3)
Unhoused	
Yes	29/128 (22.7)
No	99/128 (77.3)
Methamphetamine use	
Yes	42/128 (32.8)
GBM/transgender	24/42 (57)
Unhoused	24/42 (57)
HIV positive	23/42 (55)
No	86/128 (67.2)
Travel history (international and domestic travel)	
Yes	20/128 (15.6)
No	67/128 (52.3)
Unknown	41/128 (32.0)

*Patient age range 15–79 y, mean age 47 y. GBM, gay and bisexual man; HAART, highly active antiretroviral therapy; PrEP, preexposure prophylaxis.

*S. flexneri* infection was more common than *S. sonnei* infection among those who were living with HIV (68% vs. 43%; p = 0.008), were not unhoused (92% vs. 60%; p<0.001), did not use methamphetamines (77% vs. 54%; p = 0.006), and had not recently traveled (63% vs. 24%; p = 0.008) (Table 2). Sexual orientation and use of HAART and PrEP were not associated with *S. flexneri* or *S. sonnei* infection.

**Table 2:** Variables associated with *Shigella flexneri* and *Shigella sonnei* Infection, San Diego, California, USA, March 1, 2017–May 31, 2020*

Patient variable	*Shigella flexneri*, no. (%)	*Shigella sonnei*, no. (%)	p value
HIV positive	44 (68)	21 (43)	0.008
Sexual orientation, GBM/transgender woman	45 (85)	26 (68)	0.061
HAART use by persons living with HIV	40 (91)	15 (71)	0.065
PrEP use among HIV-negative	4 (80)	4 (57)	0.408
Unhoused	6 (8)	23 (40)	<0.001
Methamphetamine use	16 (23)	26 (46)	0.006
Travel	11 (15)	9 (20)	0.008

*GBM, gay and bisexual man; HAART, highly active antiretroviral therapy; PrEP, preexposure prophylaxis.

Of the 128 evaluable isolates, 23% were resistant to fluoroquinolones (11% of *S. flexneri* and 39% of *S. sonnei* isolates). Overall, 70% of *Shigella* spp. isolates were resistant to ampicillin (87% of *S. flexneri* and 47% of *S. sonnei* isolates), and 83% of all isolates were resistant to TMP/SMX (73% of *S. flexneri* and 95% of *S. sonnei* isolates). Only 4 (3%) isolates were susceptible to fluoroquinolones, TMP/SMX, and ampicillin; 38% were resistant to 1 antimicrobial (27% for *S. flexneri* vs. 53% for *S. sonnei*), 38% were resistant to 2 antimicrobials (62% for *S. flexneri* vs. 9% for *S. sonnei*), and 20% were resistant to 3 antimicrobials (7% for *S. flexneri* vs. 37% for *S. sonnei*) (Appendix).

Compared with persons without HIV infection, a higher percentage of *Shigella* isolates from persons with HIV infection and shigellosis were resistant to >2 antimicrobials (32% vs. 11%; p<0.001), and the rate of ampicillin resistance was higher for persons with than without HIV infection (48% vs. 30%; p<0.001). We found no difference between HIV status and fluoroquinolone or TMP/SMX resistance. Rates of *Shigella* resistance to 2 antimicrobials were higher for non-GBM/transgender persons than for GBM/transgender persons (41% vs. 3%; p<0.003).

Rates of antimicrobial resistance to 1 (25% vs. 13%; p = 0.016) or 2 antimicrobials (34% vs. 4%; p = 0.009) were higher for persons with stable housing than for those without stable housing; rates of resistance were higher for ampicillin (60% vs. 9%; p<0.001) and TMP/SMX (60% vs. 15%; p = 0.01). Compared with recent methamphetamines use, no methamphetamine use was associated with resistance to 2 antimicrobials (30% vs. 8%; p = 0.021) and higher rates of ampicillin resistance (51% vs. 19%; p = 0.042). Neither history of travel nor use of HAART was associated with increased antimicrobial resistance.

In the antimicrobial susceptibility panels at our sites, ceftriaxone and azithromycin were not commonly tested. Although isolates were not routinely tested against azithromycin, of the 36 isolates tested, 55% were resistant to azithromycin according to MIC breakpoints established by the 2022 CLSI m100 Performance Standards for Antimicrobial Susceptibility Testing for *Shigella* spp. (MIC >16 μg/mL for *S. flexneri* and >32 μg/mL for *S. sonnei*) (*21*). Azithromycin started to become more frequently tested at 1 of the sites by August 2019. Of the 128 samples analyzed, ceftriaxone susceptibility was reported for 37% of the cases without any reported resistance.

## Discussion

According to our retrospective review, *S. flexneri* and *S. sonnei* caused diarrheal disease in a large number of gender and sexual minorities who were living with HIV, persons of unhoused status, and persons who had recently used methamphetamines. Although the association between MSM and shigellosis has been reported, the rate of concomitant HIV infection and shigellosis in our study was higher than rates previously reported, including in a study of New York, New York, residents, in which 27% with shigellosis infection were living with HIV (*12*). In addition, although methamphetamine use by MSM can increase the risk for HIV and for hepatitis C virus infection (*22*,*23*), to our knowledge only a small number of reports have described an association between methamphetamine use and shigellosis, specifically among MSM (*24*,*25*). Unlike the studies that documented methamphetamine use in MSM living with HIV, we also found high rates among those who were unhoused.

Isolation of *S. flexneri* from 55% of stool specimens in our study is much higher than the national prevalence of 12.6% in 2016 reported by the Centers for Disease Control and Prevention (CDC) (*26*). Other studies have found high rates of *S. flexneri* causing shigellosis in certain populations, including in 34% of sporadic cases in a New York, New York, study (*12*) and 65% of cases from a cohort of MSM in the United States (*22*). Similarly, we found high rates of *S. flexneri* in GBM/transgender persons, persons living with HIV, and those receiving PrEP. Thus, although overall *S. flexneri* prevalence may be low in the United States, rates may be higher for certain populations, such as those reported here.

Increasing antimicrobial resistance of *Shigella* spp. has been documented globally, including in the United States. Clusters of ciprofloxacin-resistant *S. sonnei* have been reported, suspected to be associated with international travel, and 86% of isolates have been reported to be resistant (*27*). Conversely, in another study of shigellosis in MSM, all isolates were sensitive to ciprofloxacin (*28*). In our cohort, 23% of isolates were resistant to ciprofloxacin. Among MSM, decreasing susceptibility to azithromycin (MIC ≥32 μg/mL) in several *S. sonnei* outbreaks has been documented (*29*), including all isolates either resistant to (*11*) or with detected *mph*A or *erm*B macrolide resistance genes (*8*,*11*,*29*). We also detected a large number of isolates for which susceptibility to azithromycin was reduced.

Rates of antimicrobial resistance among the isolates tested were comparable to those reported in the most recent data from the CDC National Antimicrobial Resistance Monitoring System (NARMS) data (https://wwwn.cdc.gov/narmsnow), which included mostly *S. flexneri* and *S. sonnei* isolates. Among all *Shigella spp*. isolates, 55% of the isolates in our study were resistant to azithromycin compared with 54% from preliminary NARMS estimates from 2021, 23% were resistant to fluoroquinolones compared with 32% from NARMS (ciprofloxacin only), 70% were resistant to ampicillin compared with 79% from NARMS, and 83% were resistant to TMP/SMX compared with 75% from NARMS. We found no resistance to ceftriaxone in our study, which could be reflective of a smaller sample size, although of 28 isolates in 2021, only 1 (3.6%) was resistant to ceftriaxone according to NARMS data. The most recent NARMS data report a rate of 25% of resistance to ampicillin, ciprofloxacin, and TMP/SMX, which is comparable to the resistance rate of 20% to these 3 antimicrobial drugs in our study.

It is unclear why those who are unhoused in San Diego County may be at risk for shigellosis. Of note, a hepatitis A outbreak during 2016–2018 in San Diego County, which was the largest US hepatitis A outbreak in 2 decades, primarily involved persons experiencing unstable housing conditions, those who used drugs in unsanitary settings, or both (*30*). It is possible that unsanitary living conditions and illicit drug use also predispose unhoused persons to shigellosis, although further research would help clarify.

Our first study limitation is including data from only 2 medical centers in San Diego County, California, which means that our findings may not be representative of other locations within the United States or elsewhere globally. The high proportion of GBM/transgender, HIV-infected, unhoused, and methamphetamine-using persons may be unique to our setting and may not be reflective of other settings because it may not be reflective of the general population in San Diego County. Second, isolates were not routinely tested against azithromycin, which would have been helpful because this drug has traditionally been empirically used to treat shigellosis. Regardless, because most of the isolates tested were resistant to azithromycin according to the MICs, resistance to azithromycin in our setting is probably high, indicating that empiric use of azithromycin may not be beneficial. Third, given that the study was a retrospective review of EHRs, inconsistent availability of documentation of HIV status, travel, unhoused status, or methamphetamine use could have led to missing data.

In conclusion, in this retrospective study, we found shigellosis frequently diagnosed for GBM/transgender, HIV-seropositive, and unhoused persons who use methamphetamine; rates of *S. flexneri* prevalence were higher than those in national prevalence data. Overall, we found high rates of resistance to azithromycin, fluoroquinolones, ampicillin, and TMP/SMX, which were comparable to those most recently reported by CDC, except we found slightly lower resistance rates to ciprofloxacin and slightly higher resistance rates to TMP/SMX. Clinicians should remain aware of the risk for antimicrobial resistance among patients with shigellosis, particularly gender and sexual minorities, those living with HIV, persons of unhoused status, and persons who use methamphetamines.

Appendix Variables associated with *Shigella* spp. antimicrobial resistance, San Diego, California, USA, March 1, 2017, through May 31, 2020.
