# Supplementary material for: Antimicrobial-Resistant Shigella spp. in San Diego, California, USA, 2017–2020
Source: Emerg Infect Dis. 2022 Jun;28(6):1110–6. doi: 10.3201/eid2806.220131 (PMC9155871; doi:10.3201/eid2806.220131)
Supplement: Appendix — Variables associated with Shigella spp. antimicrobial resistance, San Diego, California, USA, March 1, 2017, through May 31, 2020. [file 22-0131-Techapp-s1.pdf]

Article DOI: https://doi.org/10.3201/eid2806.220131

Antimicrobial-Resistant *Shigella* spp. in San Diego, California, USA, 2017–2020

Appendix

Appendix Table. Variables associated with antimicrobial resistance, San Diego, California, USA, March 1, 2017, through May 31, 2020*

Variable	Resistant to 1 antimicrobial, no. (%)	Resistant to 2 antimicrobials, no. (%)	Resistant to 3 antimicrobials, no. (%)	Ampicillin (≥16–32, R), no. (%)	Fluoroquinolone (≥1 R), no. (%)	TMP/SMX, (≥2/38 R), no. (%)
S. flexneri	19/71 (27)	44/71 (62)	5/71 (7)	62/71 (87)	8/71 (11)	52/71 (73)
S. sonnei	30/57 (53)	5/57 (9)	21/57 (37)	27/57 (47)	22/57 (39)	54/57 (95)
p-value	0.003	<0.001	<0.001	0.001	<0.001	0.001
HIV positive						
Yes	11/114 (10)	37/114 (32)	16/114 (14)	55/114 (48)	19/114 (17)	57/114 (50)
No	37/114 (32)	12/114 (11)	11/114 (10)	34/114 (30)	11/114 (10)	49/114 (43)
p-value	<0.001	<0.001	0.596	<0.001	0.28	0.24
Sexual orientation/gender identity						
GBM/transgender	12/91 (13)	37/91 (41)	22/91 (24)	23/91 (25)	23/128 (18)	62/91 (68)
Not GBM/trans	14/91 (15)	3/91 (3)	2/91 (2)	2/91 (2)	7/128 (5)	15/91 (16)
p-value	<0.001	0.003	0.06	0.048	0.012	0.18
HAART use for Persons Living with HIV						
Yes	9/65 (14)	32/65 (49)	13/65 (20)	48/65 (74)	16/65 (25)	48/65 (74)
No	3/65 (5)	5/65 (8)	2/65 (3)	7/65 (11)	3/65 (5)	9/65 (14)
p-value	0.376	0.734	1.000	0.339	1.000	1.000
Unhoused						
Yes	17/128 (13)	5/128 (4)	7/128 (5)	12/128 (9)	7/128 (5)	29/128 (15)
No	32/128 (25)	44/128 (34)	19/128 (15)	77/128 (60)	23/128 (18)	77/128 (60)
p-value	0.016	0.009	0.603	<0.001	1.00	0.01
Methamphetamine use						
Yes	21/128 (16)	10/128 (8)	11/128 (9)	24/128 (19)	12/128 (9)	38/128 (30)
No	28/128 (22)	39/128 (30)	15/128 (12)	65/128 (51)	18/128 (14)	68/128 (53)
p-value	0.081	0.021	0.349	0.042	0.378	0.137
Travel						
Yes	9/128 (7)	7/128 (5)	3/128 (2)	12/128 (9)	4/128 (3)	16/128 (13)
No	21/128 (16)	31/128 (24)	12/128 (9)	52/128 (41)	15/128 (12)	52/128 (41)
Unknown	19/128 (15)	11/128 (9)	11/128 (9)	25/125 (20)	11/128 (9)	38/128 (30)
p-value	0.244	0.126	0.458	0.127	0.845	0.126

*GBM, gay and bisexual man; HAART, highly active antiretroviral therapy; PrEP: pre-exposure prophylaxis; R, resistance breakpoint.
